# St. Luke, the Physician

**Published:** 1916-12

**Authors:** A. Denton

**Affiliations:** 220 Formwalt St., Atlanta, Ga.


### ST. LUKE, THE PHYSICIAN.

St. Luke, writer of the Third Gospel, and the Acts of the Apostles was a physician. This fact has been conclusively demonstrated through the recent important contributions of Professor Adolph Harnack, Professor of Church History in the University of Berlin, Professor Harnack through her terse forcefulness, eminently logical mode of argument, and logical sequence having presented the argument for the traditional view in such a manner, that it would be impossible to call it a question again.

The evidence of St. Luke's membership to the profession will doubtless be of interest to all physicians.

To St. Luke's mind, the most important phase of the Lord's work was the exhibition of his curative powers· There are many incidents that he relates that emphasizes this. Luke in giving the details of the lame man cured in the third chapter of Acts, 7th verse. The story of the cure of Saul's blindness. The story of the Good Samaritan. In all these there are the interesting details that indicate medical interest on the part of the writer. St. Mark records the healing of the man with the withered hand. St. Luke adds the characteristic medical note that it was the right hand. All four Evangelists record the cutting off of the ear of the servant of the High Priest in the garden of Olives. Only St. Luke adds the information that the Lord healed it again.

To St· Luke, Jesus appeared as the great physician and healer, the Divine physician of the body as well as the soul.

Certain miracles of healing, where the blind were made to see, the lame to walk, the deaf to hear, the lepers made clean, the dead to rise again, as related by other Evangelists are retold by St. Luke from the physician's point of view, and thus the miracles have become clearer. Luke alone tells the story of the man suffering from dropsy, the woman suffering from weakness.

The fact that St. Luke was a physician is an extremely valuable one. That a physician should have been chosen as a diciple of the "Divine Physician" is an eminenetly fitting one.

A. DENTON.

220 Formwalt St., Atlanta, Ga.

